# CATS: Cas9-assisted tag switching. A high-throughput method for exchanging genomic peptide tags in yeast

**DOI:** 10.1186/s12864-020-6634-9

**Published:** 2020-03-10

**Authors:** Lisa K. Berry, Grace Heredge Thomas, Peter H. Thorpe

**Affiliations:** School of Biological and Chemical Sciences, Queen Mary University of London, Mile End Road, London, E1 4NS UK

**Keywords:** CRISPR-Cas9, Yeast, Array, GFP collection, SPA, Tag switching

## Abstract

**Background:**

The creation of arrays of yeast strains each encoding a different protein with constant tags is a powerful method for understanding how genes and their proteins control cell function. As genetic tools become more sophisticated there is a need to create custom libraries encoding proteins fused with specialised tags to query gene function. These include protein tags that enable a multitude of added functionality, such as conditional degradation, fluorescent labelling, relocalization or activation and also DNA and RNA tags that enable barcoding of genes or their mRNA products. Tools for making new libraries or modifying existing ones are becoming available, but are often limited by the number of strains they can be realistically applied to or by the need for a particular starting library.

**Results:**

We present a new recombination-based method, CATS – **C**as9-**A**ssisted **T**ag **S**witching, that switches tags in any existing library of yeast strains. This method employs the reprogrammable RNA guided nuclease, Cas9, to both introduce endogenous double strand breaks into the genome as well as liberating a linear DNA template molecule from a plasmid. It exploits the relatively high efficiency of homologous recombination in budding yeast compared with non-homologous end joining.

**Conclusions:**

The method takes less than 2 weeks, is cost effective and can simultaneously introduce multiple genetic changes, thus providing a rapid, genome-wide approach to genetic modification.

## Background

Collections of strains consisting of a set of independent isolates each with a different open reading frame (ORF) altered in the same way, are particularly useful resources for systematically testing hypotheses and for performing genetic screens. A number of these collections are genome-wide, the first of which was a deletion collection, precise knock-outs of open reading frames, created in the budding yeast, *Saccharomyces cerevisiae* [55]. This deletion collection has been immensely useful for identifying genes involved in a given process [12, 13, 16] or for the determination of how genes work together [32, 52]. Similar libraries have been made in fission yeast [24] and efforts are underway to create a deletion collection in a haploid human cell line [3]. A number of subsequent budding yeast collections have been made, including a set of GFP tagged strains to determine the localization of each cellular protein [19] and a dual epitope tag (Tandem affinity purification or TAP) collection for analysing protein levels and protein-protein interactions [11]. All of these collections have been widely used and provided important information on the function of eukaryotic cells.

Current methods in cell biology now exploit a wide array of tags for ever more sophisticated and quantitative assays on cell function, such as conditional protein degradation or single molecule mRNA detection [9, 28]. A number of studies have described new cassettes which are interchangeable with existing genomic sequences (for examples see [5, 21, 47]). However, a reliance on PCR amplification and traditional recombination methods often limits the number of strains these can be applied to. The creation of new libraries in order to test genes, mRNAs and proteins systematically using novel tags is normally prohibitively expensive for most laboratories (both financially and in time) since it requires performing potentially thousands of independent homologous targeting events. Synthetic Genetic Array (SGA) technology [51] advanced the ability to combine genetic elements from multiple into single collections, and several other technologies have since been described that use this method to allow existing tags to be switched from one to another, to meet the needs of users. Examples of the latter include The SWAp-Tag [53] and SWAT [33] custom libraries that contain an efficient recombination site in frame with each yeast open reading frame, allowing any sequence to be inserted into these ‘landing sites’. However, the library is constrained by the position of the recombination site, which determines precisely where the insertion sequence will be in relation to each open reading frame, and by the requirement for particular starting libraries. There is a need to produce a high-throughput system that can be flexible in the choice of targeting location within the genome and potentially introduce multiple genetic changes simultaneously at different locations. Such a system would allow sophisticated novel genetic tags to be used on a genome-wide scale.

The Type II CRISPR-Cas9 technology developed from *Streptococcus pyogenes* [8, 18] consists of the expression of a Cas9 endonuclease and chimeric single guide RNA (sgRNA) [23], which combines the RNA components required to form a complex with Cas9 and direct it to the corresponding site in the genome, adjacent to a Protospacer Adjacent Motif (PAM) site. In yeast, CRISPR-mediated double strand breaks (DSBs) can be repaired by two canonical repair mechanisms either non-homologous end-joining or by homology-directed repair (HDR). It is possible to use HDR to integrate novel DNA sequences into the yeast genome without the presence of an endonuclease, a feature which has led to this organism becoming an extremely useful and well-utilized tool in genetic studies. As the efficiency of HDR is increased by the presence of a DSB in the DNA [37], site-specific endonucleases, such as Cas9, have been adapted to promote HDR [18].

CRISPR-mediated genome editing has previously been applied to yeast [7, 10, 17, 31, 40, 41, 43] and has been adapted to high-throughput use [39, 45]. In this study, we utilized the endonuclease activity of CRISPR-Cas9 by targeting it to the GFP tag sequence in the genome of a library of GFP-tagged strains. By reproducing the endonuclease target site in a plasmid, we were able to convert the plasmid in vivo into a linear DNA construct containing a new tag, with homology to the GFP sequence. This linear construct is integrated into the *GFP* locus via HDR facilitated by a DSB introduced by Cas9, thereby allowing the replacement of the GFP tag with a new sequence, all requiring only one sgRNA, and avoiding the requirement for transformation of linear fragments. Cas9 endonuclease and the sgRNA can both be expressed from plasmids in *S. cerevisiae*, as demonstrated previously [7, 25], meaning all required components can be transferred into collections of strains using efficient and fast high-throughput plasmid transfer methods [38]. Novel sequences can be introduced to a collection by simple cloning of the new sequence into a plasmid, so there is also no requirement for integration of an existing array of strains with the desired constructs. We tested whether this would make it possible to efficiently create new collections of strains by swapping existing tags in one of the current yeast libraries with a novel sequence. We find that we can use Cas9-mediated cleavage of the GFP gene sequence to replace the GFP coding sequences with those encoding other peptides. We refer to this technique as CATS – **C**as9-**A**ssociated **T**ag **S**witching. The method converts around 85% of strains to the template sequence and can be used to generate a new collection at very little cost in around 2 weeks. Additionally, we report proof of principal for simultaneous introduction of two genetic changes into the genome, which potentially expands the range of tools that could be created as a library.

## Results

### Testing plasmid loss and efficiency of Cas9 cleavage in W303 yeast

Homologous recombination at a given locus is greatly facilitated by the presence of a DSB [37], since endogenous repair mechanisms are acting directly on the genome. CRISPR-Cas9 endonuclease is widely used to make targeted DSBs within genomes and therefore facilitates homologous recombination in budding yeast [7]. In these previous experiments, cleavage of the *CAN1* gene, which encodes an arginine permease, led to mutations via error-prone repair. Canavanine is a toxic analogue of arginine, hence loss of function *CAN1* mutants can be identified easily by their ability to grow on media that contains canavanine. To build upon this work, we obtained the plasmids that express *CAS9* under the control of a galactose-inducible promoter, *GAL-L* (pCas9, Supplementary Table 1) and separately the *CAN1* sgRNA under the control of a *SNR52* promoter (pCAN1-guide, [7], Supplementary Table 1).

**Fig. 1:**
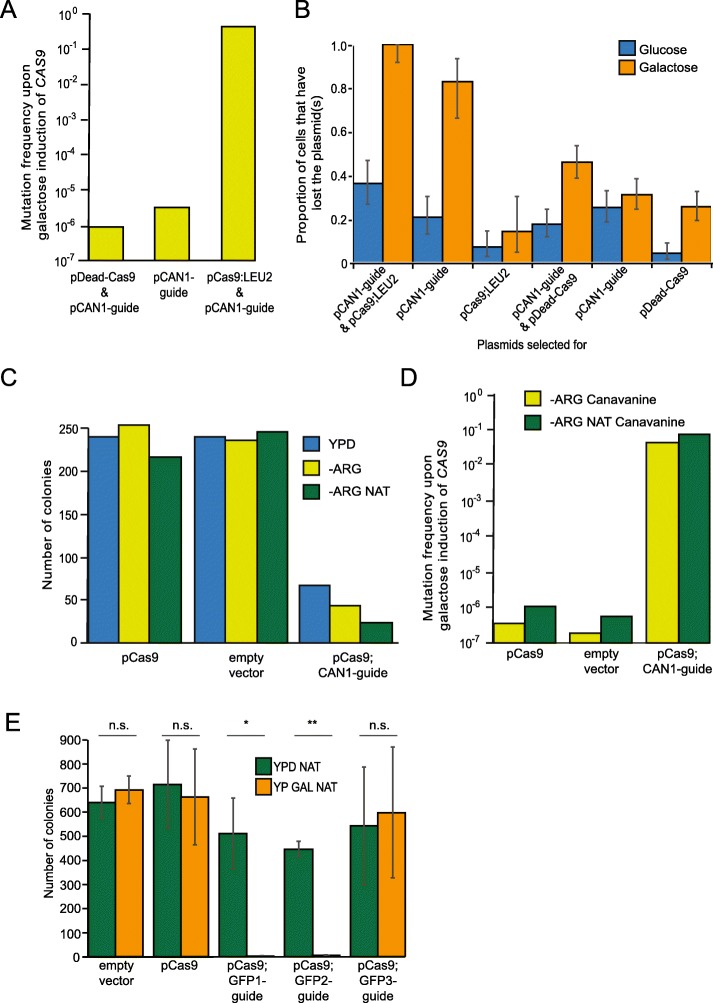
CRISPR-induced mutation frequency in *CAN1* and plasmid loss assays. **a** Frequency of mutations in the *CAN1* gene assessed by the formation of colonies on plates containing canavanine, which is toxic to *CAN1*^+^ yeast. The plasmids in strain PT141 are specified on the x-axis, and each column is a single experiment. Frequencies were calculated from the number of colonies on canavanine-containing plates compared with no drug (the media lacked uracil and leucine to select for both plasmids and also arginine to allow canavanine toxicity). **b** The rates of plasmid loss were measured, since the endonuclease complex is encoded on two separate plasmids: pCas9 and pCAN1-guide. Yeast cells (*TEF1-GFP* from the GFP collection) containing both plasmids were grown overnight with selection for both plasmids and then 500 cells were plated on medium that selects for the pCas9 plasmid (−leucine), the pCAN1-guide plasmid (−uracil) or both (−lecuine, −uracil). The resulting colonies were compared with growth without selection. The overnight growth medium contained either glucose (blue bars) or galactose (orange bars), the latter medium induces expression of the *Cas9* gene. At least one of the plasmids, pCas9 or pCAN1-guide, was lost from 10 to 40% of cells pre-grown glucose medium. This loss rate increased to nearly 100% of cells, when the *Cas9* gene was induced with galactose. The pCAN1-guide plasmid is lost more readily than the pCas9 plasmid. To determine if this loss rate was caused by the endonuclease activity, we repeated the experiment with a dead version of Cas9, which contains D10A and H840A substitutions. The plasmid loss rate on galactose was much less (30–50%) with an inactive Cas9 compared with the active Cas9, indicating that plasmid loss is associated with endonuclease function. Error bars represent exact binomial 95% confidence intervals. **c** The number of colonies observed on YPD (blue bars), −ARG (light green bars) and -ARG NAT (dark green bars, this media selects for the plasmid) media following galactose induction of a single plasmid encoding expression of both the Cas9 and guide targeting *CAN1*, and two control plasmids. Five hundred cells were plated and each column is a single experiment. **d** The frequency of mutations in the *CAN1* gene were assessed by comparing the formation of colonies on canavanine plates, with those containing no drug. The plasmids present in strain PT141 are specified on the x-axis, and each column is a single experiment. Frequencies were calculated both without selection for the plasmid (light green bars) and with NAT selection (dark green bars). **e** The effect upon viability of the different endonuclease plasmids was assessed by counting the viability of cells. One thousand five hundred *HTB2-GFP* cells (from the GFP library) that had been transformed with the plasmids stated on the x-axis were plated onto glucose (dark green bars, *CAS9* expression OFF) and galactose media (orange bars, *CAS9* expression ON), in 3 replicates (4500 cells in total). Bars represent mean and error bars represent standard deviation. **, *P* < 0.01, *, *P* < 0.05, n.s., non-significant; Welch’s two-sample t-test performed on 3 replicates

We found that approximately one third of *CAN1*^+^ cells (from strain PT141, Supplementary Table 2) which harboured both plasmids had become canavanine resistant (i.e. *can1*^−^) after induction of expression of the *CAS9* gene on galactose-containing medium (Fig. 1a). This frequency of mutation was considerably higher than that previously reported [7]. A key difference in our study compared with the previous one, is that we maintained selection for both plasmids throughout, therefore the higher rates of plasmid retention may explain our high efficiency. Consistent with this notion we tested for plasmid loss and found that the plasmids encoding Cas9 and sgRNA are lost at a high rate without selection (Fig. 1)b. We demonstrated that most of the plasmid loss is accounted for by the plasmid encoding the sgRNA, which was surprising as this is a high-copy plasmid with a 2-μm origin. We used CEN-based plasmids for all subsequent constructs.

We found that the high level of plasmid loss was related to the endonuclease activity of Cas9, since an inactive mutant version of Cas9 (pDead-Cas9) had reduced plasmid loss (Fig. 1b). We created pDead-Cas9 by introducing point mutations that encode the D10A and H840A substitutions, which inactivate the histidine-asparagine-histidine (HNH) and RuvC-like catalytic domains that are responsible for cleaving complementary and non-complementary DNA strands, respectively [23]. Persistent DSBs cause cells to arrest their cell cycle for a considerable period [42, 50], consequently, it is likely that an active endonuclease is selected against in this rapidly dividing population of cells. To minimise plasmid loss, we decided to create a single endonuclease plasmid that encodes both the Cas9 and the sgRNA guide (pCas9;CAN1-guide), as has been done previously [25]. The new plasmid also includes a nourseothricin (NAT) selectable marker gene. We chose to use drug selection because it results in toxicity for cells that do not contain a resistance gene, applying strong selective pressure to keep the plasmid. This contrasts with auxotrophic selection such as that used for the plasmids in Fig. 1b, where within a population, cells without plasmids simply arrest and may continue to replicate, for example by nutrient sharing [4].

To assess the efficiency of a single endonuclease plasmid, we repeated the *CAN1* targeting experiment using *CAN1*^+^ yeast cells (PT141, Supplementary Table 2) with pCas9;CAN1-guide. We found two features of expressing *CAS9* and the *CAN1* guide together from the single endonuclease plasmid; first, that the active endonuclease is toxic to cells, resulting in a reduced viable cell number, consistent with the presence of a persistent DSB (Fig. 1c). Second, some of the surviving colonies are able to maintain the pCas9;CAN1-guide plasmid as judged by their ability to survive on NAT (Fig. 1c). We found that 1 in 24 of the surviving colonies had mutated the *CAN1* gene, as assessed by resistance to canavanine, increasing to 1 in 13 when the NAT plasmid selection was maintained (Fig. 1d). Although this is lower than the efficiency observed using two plasmids (Fig. 1a), the far lower rate of plasmid loss justifies the use of the single endonuclease plasmid, encoding both the *CAS9* and guide sequences, for the remainder of this study.

### Targeting the endonuclease to GFP

In order to apply genomic modifications to multiple strains, we required an existing library that contains identical sequences adjacent to each open reading frame. We chose the GFP collection [19], since a subset of this library has been validated as having tags that produce detectable protein in living cells [49]. It is important to note that other tagged collections, such as the TAP-tag collection [11] should work equally well. We designed three RNA guides to GFP (Supplementary Table 1) using the ECrisp software [15] and introduced each of these guides into an endonuclease plasmid (pCas9;GFP1-guide, pCas9;GFP2-guide and pCas9;GFP3-guide).

We assessed the ability of each to cleave the genome as judged by the number of surviving colonies, since active endonuclease drastically reduces cell viability (Fig. 1c). We found that two guide sequences greatly reduced cell viability, encoded by plasmids pCas9;GFP1-guide and pCas9;GFP2-guide (Fig. 1e), which indicates that these constructs form functional endonuclease complexes. Strains that did not contain a GFP sequence were not affected for growth suggesting that the growth arrest is not caused by off-target cleavage. For the remainder of this study we used pCas9;GFP1-guide as the endonuclease plasmid.

### Initiating HDR from a plasmid sequence

**Fig. 2:**
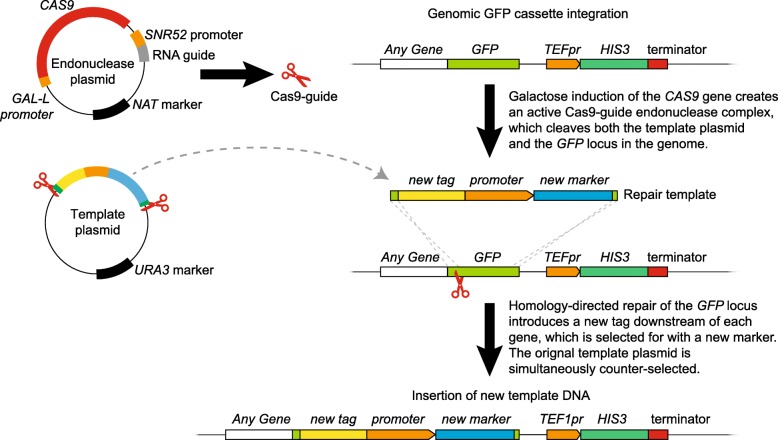
Schematic of the CATS method. CRISPR-Cas9 cleavage induces homologous repair. An *SNR52* promoter-driven RNA guide and a *GAL-L* promoter-driven *CAS9* sequence are contained in a single endonuclease plasmid conferring NAT resistance. A template plasmid, with a *URA3* marker, contains a sequence encoding a new tag and promoter-driven marker flanked by homology to the 3′ and 5′ ends of the *GFP* ORF. This template plasmid contains at either end a protospacer and corresponding PAM sequence, matching that cleaved by the expressed endonuclease. Upon galactose induction, both the genomic *GFP* ORF and the two sites in the template plasmid will be cleaved by the Cas9 endonuclease as indicated with the scissor icon. DSB-induced repair then can replace the *GFP* tag with the new template sequence

Short linear template constructs are not maintained within cells and consequently high-throughput transformation methods would be required to alter tags in a library of strains [30]. It is faster and simpler to introduce the endonuclease plasmid and template DNA via a mating-based approach [38, 52]. In order to achieve this, we asked whether we could use a plasmid to encode a template construct. We designed a sequence that includes the start of *GFP* (to provide 50 base pairs of homologous sequence) linked, in-frame, to the sequence encoding Red Fluorescent Protein (*RFP*), a new marker (the *KAN* gene encoding aminoglycoside O-phosphotransferase, which confers resistance to G418, driven by an *ADH1* promoter) and 51 base pairs of homology at the 3′ end of *GFP* (Fig. 2). In this instance we kept the HIS-MX cassette from the GFP strain intact, but it should be possible to remove this by redesigning the 3′ homologous sequence should there be a requirement to free a selectable marker. The resulting template construct will encode a new fusion protein with 16 amino acids at the C-terminus of the endogenous protein that are from the N-terminus of GFP. These amino acids provide an extended linker between the endogenous protein and the new tag, in this case RFP.

We then integrated this sequence into a plasmid, pRFP-template, and 23 bp sequences (GFP1 protospacer plus PAM sequence) were inserted on either side of the template sequence to provide recognition sites for the endonuclease product of the pCas9;GFP1-guide plasmid (Fig. 2), so that upon Cas9 induction, the linear template fragment will be generated in vivo. The protospacer and PAM sequences are aligned in opposing directions to minimize the extra sequence included in the template construct when these sites are cleaved. Since the plasmid itself confers G418 resistance, we included a *URA3* marker gene in its backbone sequence to counter-select against it after targeting. This plasmid could then be transferred in high-throughput into an array of strains using a mating-based approach [38], removing the need for multiple transformations.

**Fig. 3:**
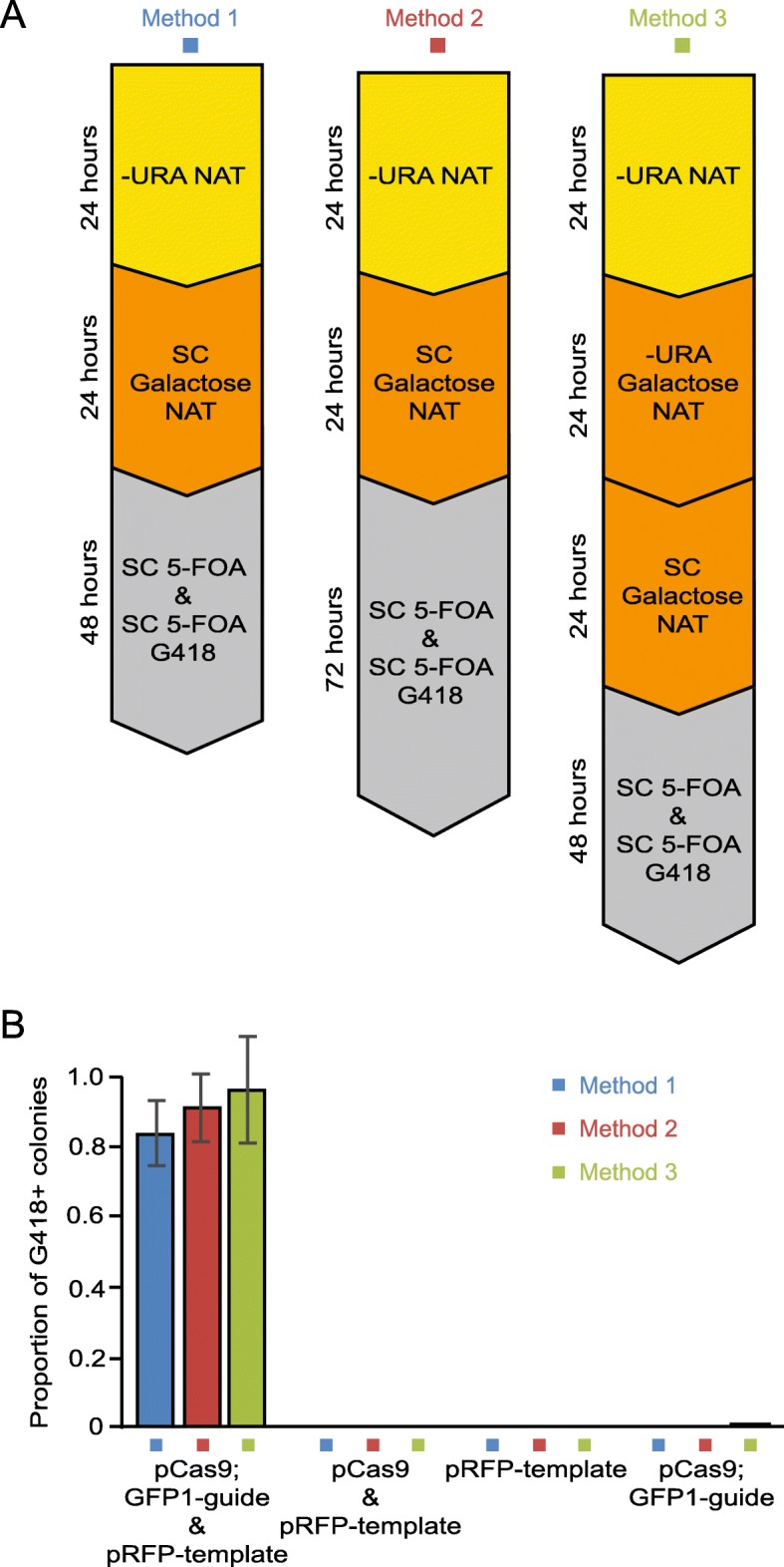
Testing three media transfer sequences for efficiency of incorporation of the template DNA. **a** Three sequences of media transfer tested on the GFP collection strain Htb2-GFP following transformation with the plasmids indicated in (**b**). Cells were washed twice with water between each media transfer, and incubation periods at each step are indicated. **b** Colonies were counted following plating of fixed numbers of cells onto SC 5-FOA and SC 5-FOA G418. Proportions represent the number of colonies formed on SC 5-FOA G418 compared to SC 5-FOA, indicating that they have integrated the RFP template plasmid, which confers G418 resistance. Results from each of the three methods in (**a**) are shown. The mean of 3 biological replicates is shown for each method and error bars represent standard deviation

To test the efficacy of this approach, we transformed both the endonuclease plasmid (pCas9;GFP1-guide) and template plasmid (pRFP-template) to a GFP strain encoding Htb2-GFP. When induced with galactose, the endonuclease complex is expressed and will cleave the three target sites – one in the *GFP* sequence in the genome and two in the template construct plasmid, thereby creating the linear template DNA with regions of homology on either side of the double strand break in the genome. We used three variant protocols to compare the efficiency of this targeting (Fig. 3a). Briefly, strains were pregrown in + NAT –URA medium to select for both the endonuclease plasmid (pCas9;GFP1-guide) and template plasmid (pRFP-template). Next, cells were switched to galactose media to induce expression of *CAS9*, and finally cells were selected on 5-fluoroorotic acid (5-FOA) to ensure that the template plasmid (pRFP-template) was lost. Targeting efficiency was judged by the proportion of resulting cells that were resistant to G418 and to the *URA3* counter-selecting drug 5-FOA. The 5-FOA selection ensures that the G418 resistance came from integration of the tempalte sequence into the genome, not from retention of the template plasmid. We found that all three methods gave a high frequency of G418 resistance (83–97%, Fig. 3b), consistent with transformation-based targeting reported previously [7].

To assess whether the resulting G418 resistant cells had converted from *GFP* to *RFP*, we isolated 18 colonies, which we tested to see if the labelled Htb2 histone was tagged with *GFP* (parental strain) or *RFP* (targeted strain). All 18 showed exclusively RFP histone labelling via fluorescence imaging. To test whether these strains had correctly targeted the *GFP* locus we amplified and sequenced the *HTB2* locus and found that 17 of the 18 had integrated the cassette correctly as illustrated in Fig. 2. In the one isolate that had integrated incorrectly, the 5′ insertion was correct, but the CRISPR target site at the 3′ end of the cassette had cut and repaired erroneously prior to gene targeting. Microhomology-based recombination at the 3′ end resulted in the cassette integrating with an extra 164 bp 3′ extension from the plasmid vector, accompanied by a 13 bp deletion from the genome.

### High-throughput tag switching

To adapt this method to a high-throughput approach, we created a new protocol that would allow the endonuclease plasmid (pCas9;GFP1-guide) and template plasmid (pRFP-template) to be transferred to an array of GFP strains using a mating-based transformation method called Selective Ploidy Ablation (SPA), [38]. In brief, SPA utilises a ‘Universal Donor Strain’ (UDS) that contains a *URA3* gene and galactose-inducible promoter (from *GAL1*) adjacent to the centromere of each and every chromosome (Supplementary Table 2). Plasmid(s) are transformed into the UDS and then this strain can be mated to an array of strains (such as the GFP collection) using high-density pinning tools. The resulting diploids are placed on galactose medium and then on 5-FOA, which first destabilises, then selects against all the chromosomes from the UDS, leaving behind a haploid GFP strain which now contains the plasmids of interest. We chose to use SPA as opposed to the SGA method [51] as it is faster for the purpose of plasmid transfer, and as both SPA and the tag switching method described above involve induction of a *GAL* promoter and then counter-selection against the *URA3* gene using 5-FOA, we reasoned that we could easily integrate these two methods for use on arrays of strains.

**Fig. 4:**
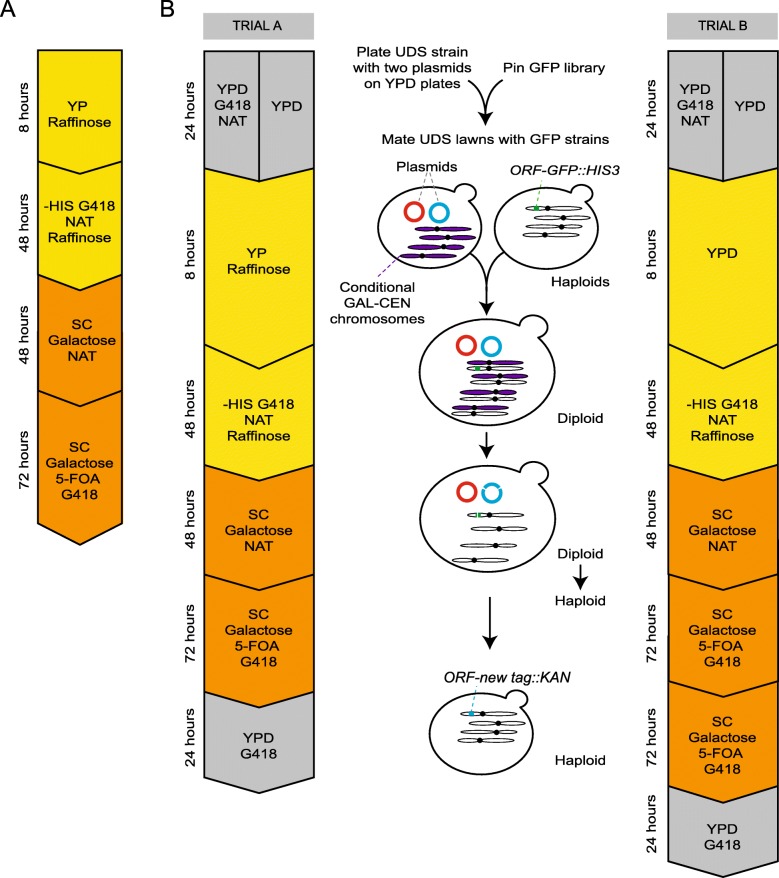
Outline of SPA-based methods for high-throughput transformation of plasmids into strains and subsequent genome editing. **a** Summary of the media transfer steps used for converting the Htb2 and Rpa49 GFP strains to the RFP template plasmid. Plasmids were pre-transformed into the UDS strain, which was mated with the Htb2 and Rpa49 strains from the GFP collection on YP-Raffinose, in the first step indicated. Subsequent media transfers select for diploid cells with both plasmids, then activate the GAL promoter-driven endonuclease, thereby beginning the replacement of the GFP tag with the template DNA. Indicated timescales refer to incubation times before transfer to the next media type. **b** High-throughput SPA method. Flowcharts indicate media transfers and incubation times on each media for trial A, which were then modified for trial B. The UDS containing the endonuclease and template plasmids was mated on YP-Raffinose with colonies from the GFP library. Selection for diploids with both plasmids was applied using -HIS G418 NAT Raffinose, before cells were transferred to galactose-containing media. This galactose induction serves two purposes: expression of the gene encoding Cas9 from the endonuclease plasmid, and selection against the UDS chromosomes through Gal-promoter mediated disruption of centromeres. Subsequent 5-FOA steps further select against the UDS chromosomes, and also against the *URA3*-containing template plasmid. The resulting colonies forming on Galactose 5-FOA G418 medium should therefore have a haploid karyotype of chromosomes originating from the GFP strains with the template DNA integrated. These strains were transferred to YPD G418 as a final selection step

To test the integration of the two methods, we initially performed a pilot experiment with two GFP strains encoding Htb2-GFP and Rpa49-GFP. The *MATα* UDS (W8164-2B, Supplementary Table 2) was transformed with both the endonuclease plasmid (pCas9;GFP1-guide) and template plasmid (pRFP-template) and mated with the GFP strains on YP Raffinose (Fig. 4a). As a control we also included an endonuclease plasmid that did not contain a guide (pCas9). After following the indicated protocol (Fig. 4a), we found that only the active endonuclease plasmid resulted in G418 and 5-FOA resistant colonies and fluorescence imaging revealed that these strains expressed RFP tagged Htb2 and Rpa29 (Supplementary Table 3).

Our aim was to be able to easily apply this method to multiple strains, therefore we mated the UDS strain containing both the endonuclease plasmid (pCas9;GFP1-guide) and template plasmid (pRFP-template) with a preassembled selection of 89 GFP strains of kinetochore-associated proteins (Supplementary Table 2), arranged in 96-array format. Colonies were then transferred between media, remaining in 96-array format, following the steps outlined in Fig. 4b Trial A. All mating and replica steps were performed using high-throughput pinning tools (Rotor HDA, Singer Instruments Ltd.), although it would also be possible to complete these steps with manual pinning tools. The 89 GFP strains from the array before media transfer were analysed by fluorescence microscopy. Of these, 68 had a detectable GFP signal, so in these strains we were able to observe whether or not our manipulated strains had converted to RFP using only fluorescence microscopy.

**Fig. 5:**
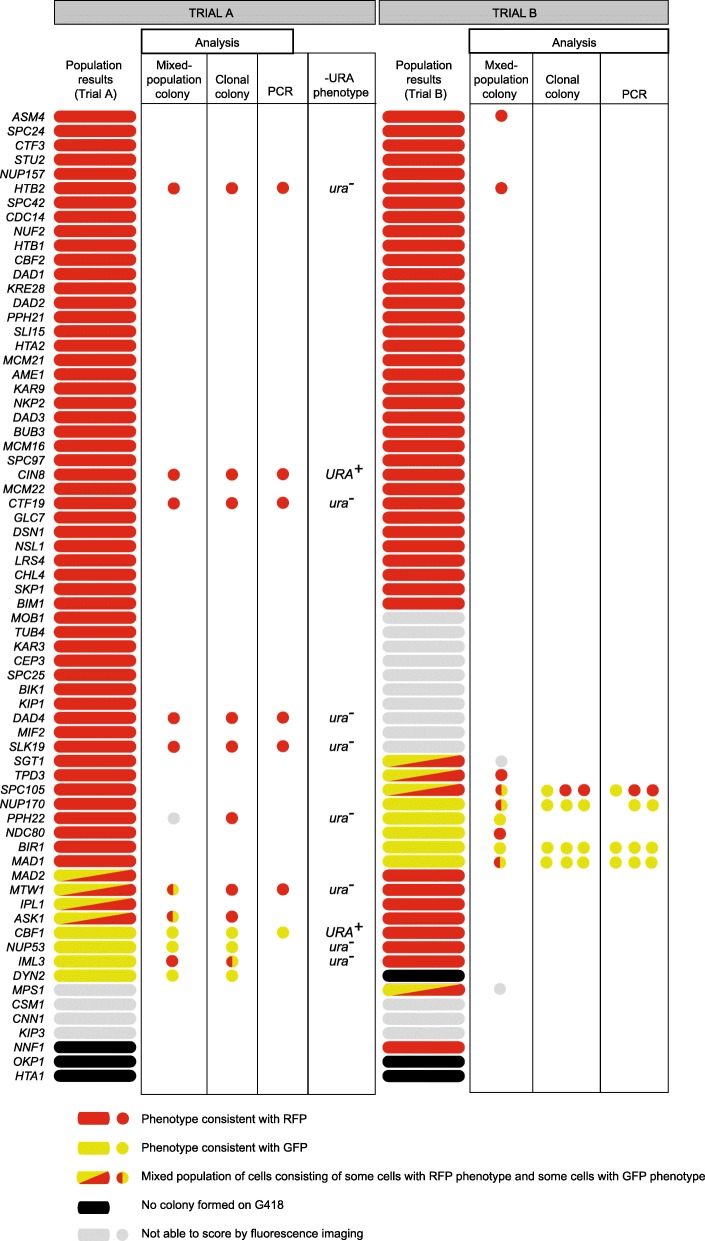
Phenotypic results from high-throughput conversion of GFP strains to RFP-G418 strains. Targeting results are indicated here for 68 strains in which a GFP signal was visible in the starting strain. Trial A and Trial B are distinguished by the separate methods indicated in Fig. 4b. The conversion from GFP to RFP is indicated in all columns by the red color. Trial A indicates microscopy results for all strains except the three indicted in black which did not form a colony on G418. Strains indicated in grey we were unable to unequivocally screen using microscopy and the remainder showed a phenotype consistent with RFP, GFP or a mixed population of both, as specified. Further analysis was then undertaken on some of the strains in Trial A, where secondary microscopy checks were performed on cells from a mixed-population colony and from a single clonal colony. The clonal colonies were then also checked for insertion of the template DNA by PCR, results indicate the detected genotype. Secondary testing for growth on 5-FOA plates shows two strains with a URA+ phenotype, indicating that the template plasmid has not been effectively counter-selected against. The methodology for Trial B was adapted to counteract this. In Trial B, cells from a mixed colony were checked with microscopy as in Trial A, then cells from mixed populations were checked again. Where possible, 3 clonal colonies were then assessed by fluorescence microscopy and PCR. The observations from each of the three colonies are indicated

Of these 68 GFP strains, the first strategy (Trial A, Fig. 4b) generated an array of 65 new strains, as 3 (~ 5%) failed to produce colonies that were resistant to both 5-FOA and G418 (*NNF1-GFP*, *OKP1-GFP* and *HTA1-GFP*) (Fig. 5, Trial A, ‘Population results’). We systematically tested the 65 new strains using fluorescent imaging, and of these we were able to unequivocally score 61 by imaging, of which 53 (~ 87%) had exclusively RFP labelling. Four strains showed a mixed population of cells, of which *MAD2-tag* and *MTW1-tag* had colocalized GFP and RFP signals within the same cell, *ASK1-tag* had some RFP and some GFP positive cells and in *IPL1-tag*, some cells were GFP, some RFP and some colocalized. A further four strains (*CBF1*-, *NUP53*-, *IML3*-, and *DYN2*-tag) had maintained the GFP expression.

High throughput methods do not generate clonal colonies, but rather a population of cells which are not clonally identical. Therefore each ‘colony’ on a high throughput plate represents a population that probably includes a number of independent targeting events. We refer to these as ‘mixed-population colonies’ to distinguish them from clonal colonies that result from the growth of a single cell. However, it is also possible that our images were captured just after the tag in the genome converted from GFP to RFP, resulting in the detection of both residual GFP-tagged protein and newly-expressed RFP-tagged protein (*MAD2-tag* and *MTW1-tag*)*.*

To further characterise the tag-switched strains, 12 mixed-population colonies were selected and their growth on –HIS and YPD G418 was confirmed. Clonal colonies were purified on YPD G418 selection and assessed again with fluorescence microscopy, from both a mixed-population colony and a single clonal colony (Fig. 5, Trial A, ‘Mixed population colony’ and ‘Clonal colony’). The genotype of selected clonal colonies was also checked with PCR where possible (Fig. 5, Trial A). Then clonal colonies were checked for growth on –URA medium as this would indicate they had retained the plasmid, explaining the ability to grow on YPD G418. In two strains, *CIN8-tag* and *CBF1-tag*, we did see growth of clonal colonies on –URA medium. We grew these strains on 5-FOA again and returned them to –URA, and they did not grow on –URA at the second attempt. Therefore we introduced an extra 5-FOA step when we repeated the experiment in Trial B, to attempt to eliminate these few strains that had G418 resistance due to template plasmid carryover (Fig. 4b, Trial B).

The repeat experiment was performed on the same 68 GFP strains, following a slightly modified protocol (Fig. 4b, Trial B). Of these 68, 3 failed to produce colonies (*DYN2-GFP*, *OKP1-GFP* and *HTA1-GFP;* Fig. 5, Trial B, ‘Population results’). It is unclear at this stage why two of the strains (OKP1-GFP and HTA1-GFP) failed to produce converted colonies in both trials. Again, we tested the 65 resulting mixed-population colonies using fluorescent imaging, and were able to score 52 of these using fluorescent signal alone, of which 43 (~ 83%) exhibited exclusively RFP labelling. Four mixed-population colonies contained both GFP and RFP cells, and 5 strains had maintained GFP expression (Fig. 5, Trial B, ‘Population results’).

11 strains from Trial B were tested for growth on G418, −HIS and -URA medium, this time growing as expected (positive on G418 and –HIS, no growth observed on –URA). The mixed-population colonies were then imaged for a second time (Fig. 5 Trial B, ‘Mixed-population colony’). Of these, there were two strains that we could not score, *SGT1*- and *MPS1-tag*. Two strains which were scored as RFP in the original trial remained RFP upon retesting (*ASM4-* and *HTB2-tag*). Two strains which were scored as mixed populations (GFP and RFP) either remained mixed (*SPC105-tag*) or were exclusively RFP (*TPD3-tag*). Finally, of five strains that were originally scored as GFP, two were confirmed to be GFP (*BIR1-* and *PPH22-tag*), two mixed (*NUP170-* and *MAD1-tag*) and one was exclusively RFP (*NDC80-RFP*) upon retrial (Fig. 5, Trial B, ‘Mixed-population colony’). This demonstrates that our tag switching method results in a small proportion of strains that do not convert from *GFP* to *RFP* despite the selection steps.

3 clonal colonies were isolated using G418 selection from strains *SPC105-*, *NUP170-*, and *MAD1-*tag, which still had both an RFP and GFP signal in the second round of imaging, and from strain *BIR1-*tag which had a consistent GFP signal in both observations. *PPH22-*tag could not be tested further due to poor growth. The clonal colonies were analysed by fluorescence microscopy and PCR to assess genotype. All of the clonal colonies from strains *NUP170-tag*, *MAD1-tag* and *BIR1-tag* (all originally scored as exclusively GFP) had GFP fluorescent signals and genotype. *SPC105-tag*, which was originally observed as having a mix of GFP and RFP signals, was confirmed as mixed from the clonal colonies; 2 isolates exhibited RFP, and 1 GFP, as judged by fluorescence imaging (Fig. 5, Trial B ‘Clonal colony’). This confirms that a small proportion of strains do not convert to RFP despite forming G418-resistant colonies. However, as this failure was observed in different strains between Trials A and B, we do not think that the failure to tag-switch a given strain is specific to the open reading frame or genomic locus.

### Genetic testing of strains that failed to convert

**Fig. 6:**
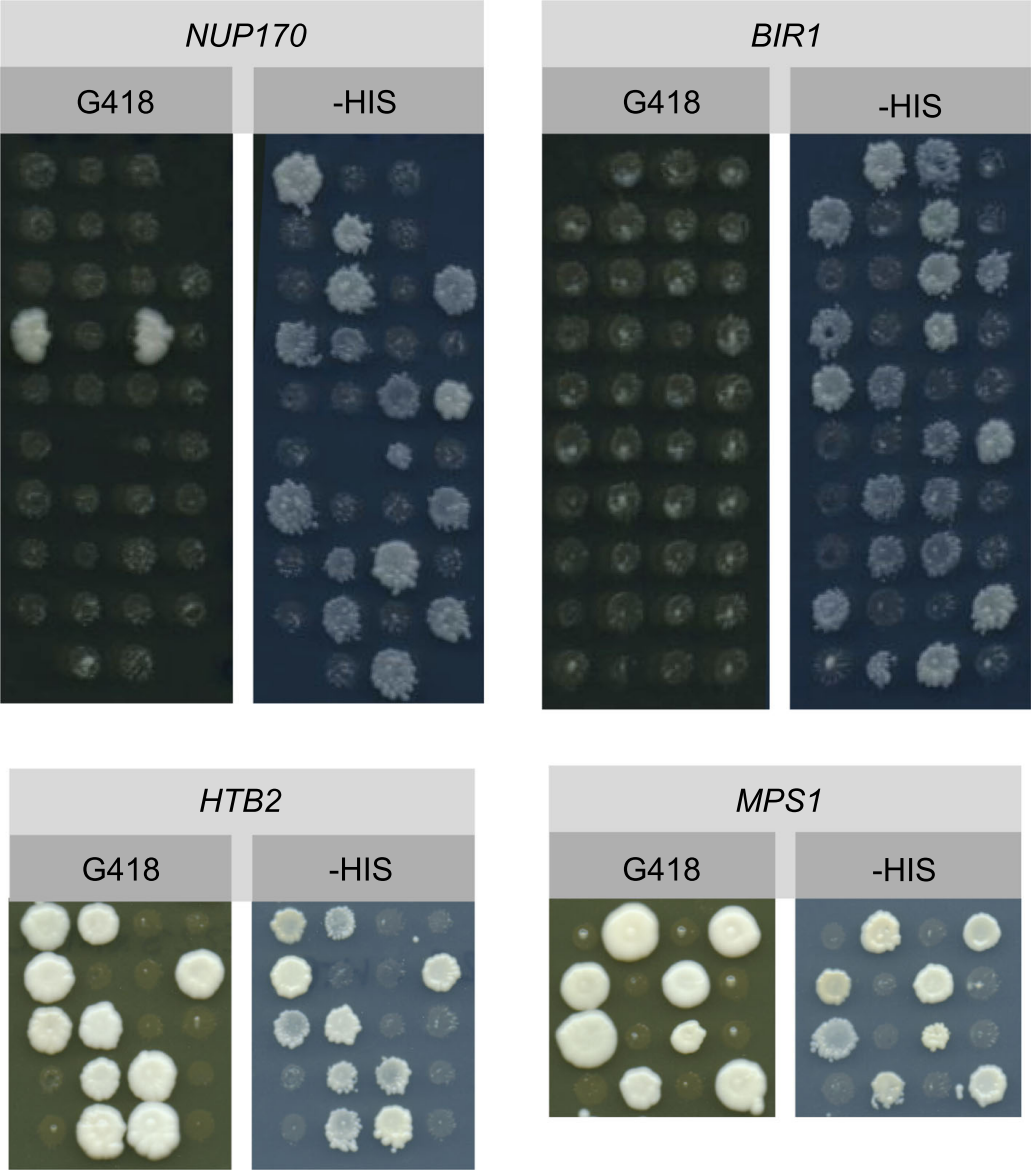
G418 resistance does not segregate correctly following meiosis in strains that retained their GFP signal. Two of the G418+ strains that retained their GFP signal (*NUP170- and BIR1-tag*) were mated with a wild-type unlabeled strain to form diploids alongside one mixed RFP/GFP strain (*MPS1-tag*) and one strain that had successfully converted to RFP (*HTB2-RFP*). The diploids were sporulated and tetrads were dissected on YPD, each tetrad was horizontally arranged and replica-plated to -HIS and YPD G418 media, then scored for growth as indicated in Supplementary Table 4. Images show the subsequent colony formation of replicated spore colonies

The protocol outlined in Fig. 2 should result in all colonies formed on G418 containing the RFP tag. We therefore wanted to investigate how strains were arising that remained GFP, while also becoming G418 resistant. One possibility is that the template construct is inserting elsewhere in the genome. Alternatively, the template plasmid could lose or mutate the *URA3* gene. To distinguish between these two possibilities, we followed the segregation of the G418 and *HIS3* markers through meiosis, since the two should be linked if the tag has correctly switched (Fig. 2). One tag-switched strain from Trial B, which had correctly converted (*HTB2-tag*), one mixed strain (*MPS1-tag*) and two which had remained GFP (*NUP170-* and *BIR1-tag*) were crossed with a wild type *MAT*alpha strain, E223, sporulated and tetrads dissected (Fig. 6, Supplementary Table 4). The non-mendelian segregation of the G418 marker gene from diploid strains heterozygous for the *NUP170-tag* and *BIR1-tag* indicates that the genetic source of G418 resistance observed in these strains is not carried on a standard chromosome. One tetrad from the *NUP170-tag* strain formed two G418 resistant colonies but these fail to segregate with the *HIS3* marker. In contrast, G418 resistance cosegregates with the *HIS3* marker in the *HTB2-tag* and *MPS1-tag* strains (Supplementary Table 4, Fig. 6). These data demonstrate that the template constructs, or at least the *KAN* marker gene, in the *NUP170-tag* and *BIR1-tag* strains has not integrated into the genome and is instead propagating independently, possibly by mutation of the *URA3* gene in the template plasmid. Our data do not rule out that in some cases the template DNA will be incorporated non-specifically into the genome.

### Converting GFP strains to different tags

**Fig. 7:**
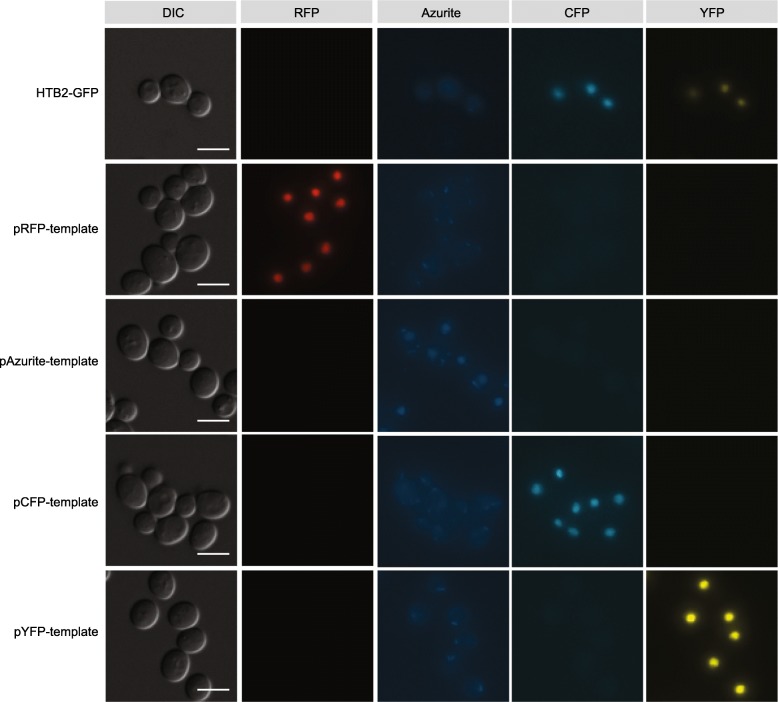
Conversion to different fluorescent tags using variants of the template plasmid. The strain encoding Htb2-GFP was tested with 4 different template construct plasmids, specified for each horizontal panel, each encoding a different fluorescent tag combined with ADH1 promoter-driven Kanamycin. Strains were then checked with fluorescence microscopy, and signals in the DIC, RFP, Azurite, CFP and YFP channels are shown. Scale bars represent 5 μm. GFP appears in both the CFP and YFP channels due to the use of a single band pass filter. It remains distinguishable from the CFP and YFP signals however as each of these appear in only one channel

To test the flexibility of the system, new template plasmids were made that follow the same principle of replacing the *GFP* tag in the GFP collection, but use different fluorescent tags to the *RFP* tested thus far. To this purpose, we replaced the *RFP* sequence in plasmid pRFP-template with sequences encoding different fluorescent tags azurite, cyan fluorescent protein (CFP) and yellow fluorescent protein (YFP), maintaining the *ADH1p-KAN* sequence that follows. This allowed us to repeat the experiment in Fig. 4 A with plasmids pAzurite-template, pCFP-template and pYFP-template (Supplementary Table 1). Final colonies were transferred to YPD G418 before being checked by fluorescence microscopy (Fig. 7) and PCR, followed by sequencing. All colonies checked had the correct genotype and the PCR analysis showed that the switched tag had inserted as designed.

### Off target cleavage

Yeast has a compact genome (12 × 10^6^ base pairs) with relatively few repeats and consequently gene targeting by meganucleases, such as Cas9, are rarely thought to generate off-target cleavage (see [27, 41, 46]). A single DSB in yeast causes a significant delay in cell cycle progression and therefore would be strongly selected against (Fig. 1e and [42, 50, 54]). It is possible that off-target cleavage occurs sporadically and is rapidly repaired (perhaps erroneously) to create off-target mutations. However, assays using meganucleases in yeast suggest that if rapid cycles of cleavage and repair do occur, they result in accurate repair of the break site, that can be re-cleaved by the endonuclease, hence promoting persistent cell cycle arrest (Fig. 1e and [26]). Nevertheless, we assessed the yeast genome for sites that are most similar to the GFP target site within the genome, and then assessed which of these had a PAM site 3′ of the sequence. We identified three sites that had minimal mismatches, consistent with off-target sites [56]. These sites are within the *YLL012W*, *YJR102C* and *YOR131C* genes. We amplified and sequenced these regions in 4 GFP strains (GFP-tagged alleles of *YDL088C*, *YDR010W*, *YHR030C*, *YLL003W*) and in 3–4 isolates of a strain that contains no GFP sequence (BY4741), all of which had been targeted using the pYFP-template plasmid. We were unable to identify any mutations in any of these regions (Supplementary Figure 1). While this analysis is limited, collectively our data and that from other laboratories suggest that off-target effects are rare when using the GFP guide. It should be noted that other guides may produce off-target effects. Furthermore, it is possible that if protein tags alter the function of their attached proteins, they will likely impose strong selection for compensatory mutations elsewhere within the genome, as has been noted for the yeast deletion collection [48]. This effect may be a greater source of mutations within targeted strains than off-target cleavage effects.

### Multiple targeting events

For some applications, such as the introduction of the auxin-inducible degron [34, 35], it would be useful to make more than one genomic change at a time. The auxin degron requires a tag introduced onto each protein but also the presence of the *TIR1* gene encoding an auxin-dependent substrate binding protein of the Skp, Cullin, F-box E3 ubiquitin ligase complex (SCF), that conditionally targets the tagged proteins for degradation upon auxin treatment. We therefore decided to test whether our method could perform two changes simultaneously, whilst still only requiring two plasmids.

**Fig. 8:**
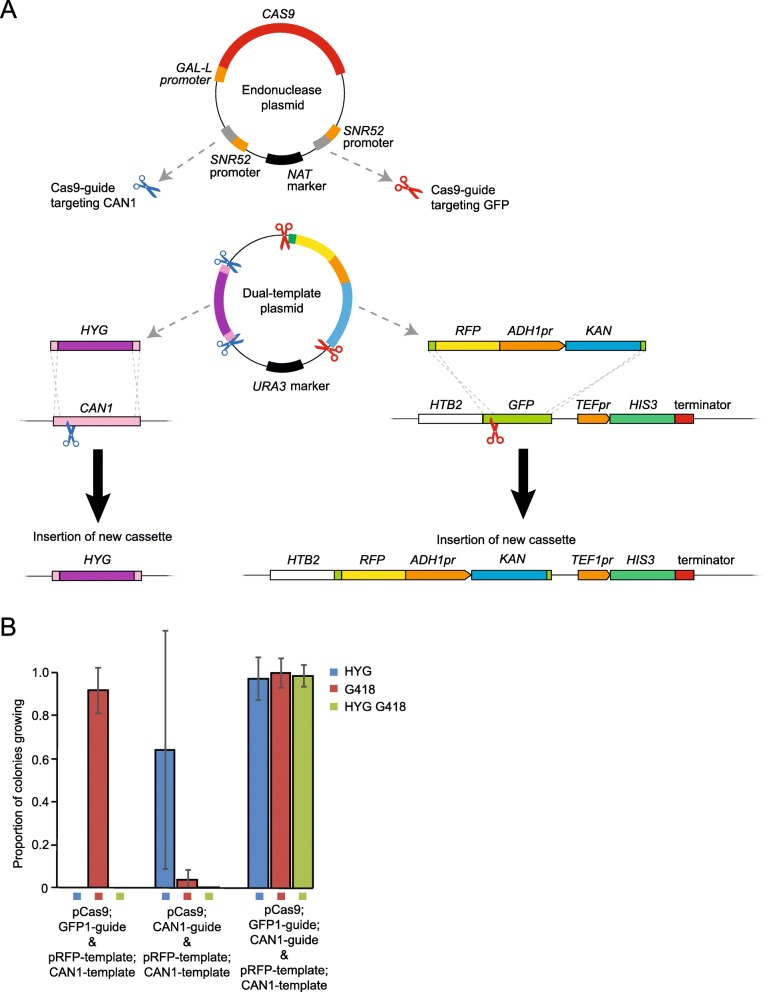
Simultaneous conversion to dual targeting constructs using two endonucleases. **a** Schematic of the targeting process with two different DNA templates, to replace two regions of the genome. The endonuclease plasmid contains a *GAL-L* promoter-driven *CAS9* gene and two *SNR52* promoter-driven sgRNA sequences, one targeting *GFP* and the other *CAN1*. The dual-template plasmid contains two template sequences, one to replace *CAN1* with *HYG*, and the other to replace *GFP* with an *RFP-ADH1 promoter-Kanamycin* sequence, as demonstrated in Fig. 2. Each is flanked by homology to the endogenous gene, and by the relevant protospacers, so that each endonuclease complex will cut both the genome and the plasmid template sequence, inducing HDR into both loci. **b** Proportions of converted strains from the dual targeting experiment illustrated in (**a**). *CAN1* targeting confers HYG resistance, and conversion from *GFP* to *RFP* confers G418 resistance. The strain Htb2-GFP contained the dual-template plasmid alongside an endonuclease plasmid encoding both the *GFP* and *CAN1* guides, or each of these individually, as indicated. The proportions are calculated against the number of colonies growing on YPD without selection, and shown here is the mean and standard deviation (error bars) of 3 biological replicates

For this purpose, we made a new endonuclease plasmid, pCas9;GFP1-guide;CAN1-guide, which contains the *GAL-L* promoter-driven *CAS9*, sgRNA targeting *GFP* and a further sgRNA targeting *CAN1*, using the same target sequence as in pCas9;CAN1-guide. The guides were engineered on either side of the *NAT* selectable marker (Fig. 8a) to select against “pop-out” recombination events that might occur between the identical guide promoter or terminator sequences in the plasmid.

We also incorporated a second sequence into the template plasmid, which contains a Hygromycin (*HYG*)-resistance gene flanked either side by ~ 50 bp homology to either end of the *CAN1* gene. This whole sequence is then flanked by *CAN1* protospacer sequences, including PAM sequences, in opposite directions in a similar design to that used for the *GFP* template construct, creating endonuclease target sites for the CAN1-guide endonuclease. When incorporated into the genome, this second template construct should confer resistance to both Hygromycin B and canavanine due to the replacement of *CAN1* with *HYG*.

In order to test this, we transformed the pRFP-template;CAN1-template plasmid into cells encoding Htb2-GFP along with either the pCas9;GFP1-guide;CAN1-guide plasmid, pCas9;GFP1-guide or pCas9;CAN1-guide. Upon galactose induction of *CAS9* we were able to quantify the number of incorporations of either template sequence by selecting on HYG for the *CAN1* deletion and/or G418 for the *GFP* to *RFP* conversion. We observed that in cells containing the pCas9;GFP1-guide;CAN1-guide and pRFP-template;CAN1-template plasmids in combination, the efficiency of conversion to both HYG and G418 resistance was 98.5% (Fig. 8b). In order to confirm the insertions from the targeting plasmids, we then selected 18 colonies from these strains on YPD HYG G418 and tested for growth on –ARG Canavanine before also scoring fluorescent signal under the microscope, and all were found to give the expected *can1*^−^ and RFP phenotypes (Supplementary Table 5).

In this experiment there were examples of gene targeting in the absence of one of the two guide sequences – most prominently the presence of G418+ colonies conferred by the *GFP* locus replacement, but achieved with the pCas9;CAN1-guide plasmid. We predict that this background conversion is due to the presence of the linearized template plasmid once the template construct of choice has been cut at the two engineered CRISPR sites. This remaining, now linear, construct still includes the *GFP* template sequence, so although it has not been cut at the flanking target sites which we have shown recombine at high frequency with the presence of a DSB in *GFP*, it is still capable of recombining at a lower frequency into the genome. The frequency of either targeting event remains much higher however when accompanied by the appropriate DSB (Fig. 8b).

## Discussion

In this study, we introduce CATS, a novel method that uses CRISPR-Cas9-mediated genome cleavage to promote replacement of endogenous DNA sequences in order to create new libraries of strains from existing collections. Previous studies have succeeded in tagging many of the ORFs in *S. cerevisiae* with, for example, GFP [19] or dual epitope tags [11], and collections also exist with gene deletions [55] or temperature sensitive alleles tagged with a selectable marker [29]. These collections have enabled many novel biological discoveries and are widely used, but as the variety of potential tags increase, there is a need to be able to introduce them into arrays of strains, without the requirement for independent targeting events with individual constructs, which is costly both in time, and financially.

Previous studies have demonstrated highly efficient genome engineering using CRISPR in yeast, including the insertion of entire pathways or disruption of multiple genes [1, 2, 17, 20, 22, 31, 41]. The use of CRISPR-Cas9 increases the efficiency of HDR, both due to the induction of a DNA damage response [37], and also the cell cycle arrest or lethality caused by unrepaired DSBs, which adds a selection pressure for cells that have undergone HDR. However, there remains a reliance on the transfer of linear constructs into cells. In this study, we integrated our template construct, which contains homology to GFP, into a plasmid flanked by GFP protospacers, so it can be transferred into cells alongside a second plasmid encoding the endonuclease components using high-throughput transfer methods [38], and cut in vivo to form a linear construct. CRISPR-mediated HDR remains highly efficient using this method as demonstrated in an individual strain, with 97% conversion using a single construct, or 98.5% conversion for a double targeting event. With such high efficiency, it could be possible to remove the selectable markers, allowing for the development of more complex strains by removing the limitation of the number of selectable markers available. We show proof of principle for targeting both the *GFP* gene (in multiple genomic locations) and the *CAN1* gene, at its endogenous location. In principle, the method could be used to target other tags, such as the tandem affinity purification tag, which was constructed as a genome-wide library in yeast [11]. We cannot predict that all genomic loci will provide such high levels of gene targeting.

Other high-throughput methods are available for diversifying collections of strains, for example, [33] outline the SWAp-Tag method, in which they have constructed a library containing an acceptor module at the C-terminus of 5661 strains. A library is also available containing N-terminal acceptor modules [53]. Once a laboratory has this collection of strains, the method of switching the acceptor sequence to a donor sequence follows the SGA procedure [51]. CATS, in contrast to SWAp-tag, does not require a specific starting library as it can be designed to target any collection in which there is a consistent protospacer sequence, also having the flexibility of being able to introduce any new sequence, in theory, by cloning the desired sequence into a single template plasmid. Plasmid transfer via SPA is also faster and requires less resources (due to a reduced number of media transfer steps) than a meiosis-based protocol.

There have also been previous attempts to edit pre-existing libraries, such as the CRISPR-UnLOCK method [39] in which CRISPR-mediated HDR on existing strains is used. However, there is again a reliance on linear donor constructs, limiting its ability to be applied to collections. We have demonstrated that CATS can easily be scaled to multiple strains without a corresponding increase in workload.

Our method is however limited by the conversion rate of approximately 85% across an array of strains. We investigated the strains which did not convert to the RFP when using the pRFP-template construct in our two high-throughput trials, and for the 68 strains we used which had a visible GFP signal, it was feasible to do this assessment quickly and systematically using fluorescence microscopy, demonstrating the practicality of our method for high-throughput application. First, we found that in two strains in Trial A, G418 resistance was conferred by likely retention of the template plasmid. This was addressed by the introduction of a second 5-FOA step in the methodology for Trial B. We also discovered that in some strains, both GFP and RFP phenotypes can be observed, likely due to the mixed population nature of our screening method. When tested further, two of the strains that had a GFP fluorescent signal but were G418-resistant were found to lose this resistance by passing through meiosis. This indicates a failure in the integration of the Kanamycin gene. Further observations on strains that did not appear to be correct in the first instance sometimes showed mixed populations, meaning the recorded success rate (conversion to RFP) of 87% in Trial A and 83% in Trial B could be increased if researchers are willing to further screen and purify these strains. We could also potentially improve the targeting efficiency by elevating the levels of template construct, for example by replacing the centromeric template plasmid with a high-copy 2-μm plasmid. Additionally, mixed populations can be subject to repeated selection steps to enrich for correctly-targeted strains. Further optimisation of the method could allow application to genome-wide arrays.

Our method also presents the risk of endonuclease plasmids persisting in the population further to the creation of new strains. However, we have shown that endonuclease plasmids are lost at a high rate from the cells if not selected for, so the risk of persistence should be low. Furthermore, the *CAS9* expression is controlled by a *GAL-L* promoter [7], so if the plasmid should persist, the *CAS9* gene will not be expressed as long as the cells are maintained on glucose.

To our knowledge, this is the first study to use plasmid-based donor DNA in combination with CRISPR to facilitate the fast and cheap conversion of a multitude of existing libraries to other tags, markers or genetic constructs. We were able to achieve highly efficient genome engineering with either one or two constructs, contributing to the range of tools available to the yeast community for making increasingly complex strains with a reduced need for selectable markers. The method can potentially be applied to insert any donor sequence into any starting library, enabling researchers to select from a wide range of peptide tags for easy insertion into an array of their choosing. The CATS method is designed for a one-step tag switching process. When multiple tags are changed, these are done simultaneously. The method is currently not designed for iterative, multi-step, tag switching.

## Conclusions

The CATS method (Cas9-Assisted Tag Switching) reported here is a novel method that rapidly switches genetically encoded tags using homologous recombination in yeast. The method employs a guided Cas9 endonuclease-mediated double-strand break coupled with a linear DNA template that is self-generated in vivo using the same endonuclease. The majority of the resulting strains encode a new genetic tag, allowing arrays of new strains to be generated quickly and cheaply. The targeting strategy is flexible since it can be applied to any sequence in the genome.

## Methods

### Standard methods

Yeast cultures were grown at 30 °C in YPD (1% yeast extract, 2% peptone, 2% glucose) or amino acid dropout medium (0.7% yeast nitrogen base, various amino acids, 2% glucose or to allow drug addition, 0.2% yeast nitrogen base excluding ammonium sulfate, 1% monosodium glutamate, various amino acids, 2% glucose). For plates, 2% agar is added, and in any media the 2% glucose was substituted for 2% raffinose, or 2% galactose supplemented with 1% raffinose to change the carbon source. Standard yeast growth conditions were generally used unless otherwise stated [44]. Media additions were added at the following concentrations: G418, 300 μg/ml; hygromycin B, 200 μg/ml; nourseothricin (NAT), 100 μg/ml and canavanine, 60 μg/ml (in –ARG media). Yeast cell densities in culture were measured using a hemocytometer.

Transformation of DNA into yeast followed standard lithium acetate methods [14]. Plasmid transformations into *E. coli* strains DH10B (Thermofisher Scientific), XL10-GOLD Ultracompetent (Agilent Technologies) or NEB Stable Competent *E. coli* (New England Biolabs) were performed following the supplied protocols and cultures were grown in standard LB media with the addition of Ampicillin at a final concentration of 50 mg/ml.

Plasmids were assembled using either gap repair cloning techniques, or NEBuilder HiFi DNA Assembly Master Mix (New England Biolabs). All PCRs in this study used DreamTaq Green PCR Master Mix (ThermoFisher Scientific) or Q5 polymerase (New England Biolabs). All primers in this study were obtained from Sigma-Aldrich Company Ltd. and plasmid sequences were validated using Sanger sequencing (GeneWiz UK, The Francis Crick Institute, or Source BioScience).

### Fluorescence microscopy

Cultures for microscopy were grown overnight at 23 °C, to optimise folding and maturation of the fluorophores, in relevant dropout or drug selectable media plus 100 mg/L adenine. Cells were imaged either directly from overnight cultures, or by embedding the cells into 0.7% low melting point agarose, with a Zeiss Axioimager Z2 microscope (Carl Zeiss AG, Germany), using a 63 × 1.4NA oil immersion lens. Illumination was from a Zeiss Colibri LED illumination system (GFP = 470 nm, YFP = 505 nm, RFP = 590 nm, CFP = 445 nm and Azurite = 385 nm). Bright field contrast was enhanced with differential interference contrast (DIC) prisms. The resulting light was captured using a Hamamatsu Flash 4 Lte. CMOS camera containing a FL-400 sensor with 6.5 μm pixels, binned 2 × 2. Exposure times were set to ensure that pixels were not saturated and were identical between control and experimental images. All images were acquired using either Axiovision or Zen software from Zeiss, then images to be shown in the publication were prepared using Icy [6] and Illustrator (Adobe).

### Calculating mutagenesis frequency

Plasmids p426-SNR52p-gRNA.CAN1.Y-SUP4t (herein referred to as pCAN1-guide) and p415-GalL-Cas9-CYC1t (herein referred to as pCas9) were a gift from George Church [7]. Inactivating mutations D10A and H840A [23] were introduced in the *CAS9* gene to create pDead-Cas9, using a QuikChange Lightning Multi Site-Directed Mutagenesis Kit (Agilent Technologies) with primers CAS9-D10A-F (sequence 5′-CAAGAAGTACTCCATTGGGCTAGCTATCGGCACAAACAGCGTCGG-3′) and CAS9-H840A-F2 (sequence 5′-CTCTCCGACTACGACGTGGACGCAATTGTGCCCCAGTCTTTTCTC-3′).

Plasmid pCAN1-guide was transformed into strain PT141 in combination with pCAS9, pDead-Cas9 or alone, and selected for on –URA –LEU or –URA for the single plasmid strain. Cultures were set up in the relevant glucose dropout media. The following day, cells were washed twice with water and diluted to an O.D. of 0.3 in galactose media, maintaining the dropouts for plasmid selection. After overnight incubation, cells were again washed twice with water and counted, and 1500 cells were plated on control plates (−URA –LEU –ARG for double plasmids, −URA –ARG for single plasmid). A further 1.1 × 10^7^ cells were plated onto –URA –LEU –ARG canavanine media from the double plasmid, or 1.1 × 10^6^ cells from the single plasmid culture. After 2 days of growth colonies were counted, and the frequency of mutations was calculated by comparing colony numbers on canavanine-containing plates to those on plates with no canavanine.

### Plasmid loss assay

Plasmid pCAN1-guide was transformed into the strain encoding Tef1-GFP in combination with either pCas9 or pDead-Cas9. Cultures were set up in –URA –LEU dropout media with glucose and galactose in separate cultures. Following overnight growth, cells were counted and 500 were plated on –URA –LEU, −URA and –LEU dropout media with the carbon source maintained from that in culture. Glucose cultures were also plated on YPD, and galactose cultures on YP GAL for comparison. After 3 days’ growth, colonies were counted and plasmid loss was calculated as number of colonies on glucose or galactose containing dropout medium as a proportion of the number of colonies on YPD or YP GAL respectively.

### Making a single plasmid to express Cas9 and guide

pCas9 was used as a template to amplify the *GAL-Lp-CAS9* sequence which was cloned into multiple plasmids (see Supplementary Table 1 for details), using an empty NAT-selectable vector [36] for insertion of both the *CAS9* and sgRNA sequences from pCas9 and pCAN1-guide respectively. This allows the expression of both components of the endonuclease from the same plasmid, with a NAT selectable marker, as the drug selection should increase the selective pressure to retain the plasmids. In all plasmids in this study, expression of *CAS9* is inducible via a *GAL-L* promoter, an attenuated *GAL1* promoter, to limit the potential toxicity of Cas9 activity [7].

### Testing endonuclease efficiency of components expressed from the same plasmid

pCas9;CAN1-guide, pCas9 and the empty vector were transformed individually into strain PT141 and selected for on YPD NAT. Cultures were set up in YPD NAT and grown overnight at 30 °C. Cells were then washed twice with water and diluted into 5 ml YP GAL NAT, then left to grow at 30 °C for a further 24 h. Cells were again washed twice with water, resuspended and 10x dilutions were performed, so that cell density could be calculated. Five hundred cells were plated onto –ARG and –ARG NAT glucose dropout media and YPD. 1.1 × 10^7^ cells were plated onto –ARG canavanine and –ARG NAT canavanine glucose media from the pCas9 and empty vector control cultures, or 1.1 × 10^5^ cells from the pCas9;CAN1-guide culture. After 2 days of growth colonies were counted, and the frequency of mutations was calculated by comparing colony numbers on –ARG canavanine to –ARG, and on –ARG NAT canavanine to –ARG NAT, following correction for the different numbers of cells plated.

### Testing the efficiency of different GFP RNA guide sequences

sgRNA targeting sequences within the *GFP* gene were designed using the E-CRISP website [15] and assembled into the sgRNA sequence from pCAN1-guide [7], forming plasmids pCas9;GFP1-guide, pCas9;GFP2-guide and pCas9;GFP3-guide. For guide sequences, see Supplementary Table 1.

Overnight cultures of the GFP collection strain, *HTB2-GFP*, containing plasmids pCas9;GFP1-guide, pCas9;GFP2-guide and pCas9;GFP3-guide, with pCas9 and the empty vector as controls were inoculated in YPD NAT medium, in 3 biological replicates. The following day, 500 and 1000 cells were plated from each culture onto YPD NAT and YPD Gal NAT. Colonies were counted after 3 days’ growth, highlighting toxicity associated with plasmids pCas9;GFP1-guide and pCas9;GFP2-guide. Further experiments used only pCas9;GFP1-guide.

### Marker switching with a template plasmid

Figure 2 outlines the marker switching method. Cas9 and an sgRNA sequence targeting the GFP were expressed from a single NAT-selectable plasmid, pCas9;GFP1-guide, driven by GAL-L and SNR52 promoters respectively. This plasmid expresses the Cas9 endonuclease and sgRNA complex which targets and cleaves a specific site in the *GFP* sequence in the genome of any GFP collection strain [19]. A second plasmid, pRFP-template, contains the template DNA, which contains a new tag, promoter and new marker, flanked by (~ 50 bp) sequences homologous to the termini of the *GFP* sequence. On either side of this, there is a targeting site and PAM sequence originally from the *GFP* ORF to match the sequence targeted by the sgRNA. These are aligned in opposing directions to minimize the extra sequence included when these sites are cleaved by the endonuclease complex. The selectable marker for this plasmid is *URA3*, so that the plasmid is also counter-selectable using media containing 5-FOA.

To initially test the plasmid-cleaving method, the following plasmids were transferred into strain *HTB2-GFP*: pCas9;GFP1-guide with pRFP-template, pCas9 with pRFP-template, pRFP-template alone and pCas9;GFP1-guide alone. Clones were isolated in 3 biological replicates and set up in glucose culture conferring –URA NAT selection. Transfer to galactose-containing media then followed 3 protocols, outlined in Fig. 3a. In Methods 1 and 2, cells were transferred to SC galactose NAT, whilst in Method 3, a –URA galactose NAT step occurred before this, thereby increasing the time that the cells spent under URA selection. All transfer between media included washing of the cells twice with water. Cells were plated on SC 5-FOA and SC 5-FOA G418 glucose plates in order to identify colonies where the G418 resistance gene had been incorporated into the genome. Colonies were counted after the time specified in Fig. 3a. Eighteen of the G418 positive colonies (6 from each method) were purified to clonal colonies and insertion of the template DNA was confirmed with microscopy and PCR.

The selection method was then adapted to include transformation of the plasmid, to allow for high-throughput conversions. Plasmids were transformed into a universal donor strain (UDS, W8164-2B) and these cells were mated with cells from the GFP strains encoding Htb2-GFP and Rpa49-GFP on YP-Raffinose, to allow the formation of diploids. Subsequent transfers to –HIS G418 NAT Raffinose, SC Galactose NAT and SC Galactose 5-FOA were done using a toothpick in the timescales outlined in Fig. 4a, and cells from the final plate were checked with fluorescence microscopy for RFP conversion.

### Marker switching via SPA

Eighty-nine GFP strains of kinetochore-associated proteins and 7 control strains were arranged in 96-array format using a Rotor HDA (Singer Instruments Ltd.). An identical sequence of media transfer to that in Fig. 4a was followed, outlined in Fig. 4b (Trial A), and all transfers were performed using the Rotor HDA. All of the original GFP strains and those from the YPD G418 plate were checked by fluorescence microscopy. The results from this were produced by assessing the signal live as well as from an image captured from each strain then reaching a consensus. Strains have been removed from the analysis if no GFP signal was visible in the original GFP strain as this removes the possibility of assessing conversion by microscopy.

Twelve strains were then selected and purified on –HIS and YPD G418 to check growth. Colonies from the YPD G418 selection were assessed again with fluorescence microscopy, from both a mix of colonies and a single isolate, and single isolate genotypes were also checked with PCR where possible. As before, signals were assessed live as well as from an image captured from each strain, then results were reached from the consensus. Cells were also checked for colony formation on –URA plates. Those that grew were grown on 5-FOA plates and returned to –URA where they did not grow on the second attempt.

A second sequence of media transfer was designed for repeating the experiment, based on the identification from Trial A that the inclusion of a second 5-FOA step would likely reduce the number of false positives. This sequence is outlined in Fig. 4b (Trial B). All transfers were again performed using the Rotor HDA and the strains were the same as used in Trial A.

Twelve strains from Trial B were tested for growth on YPD G418, −HIS and –URA plates and strains were then checked again from the YPD G418 plate with microscopy. Strains that persistently showed GFP or mixed fluorescent signals were purified and single colonies were assessed by fluorescence microscopy and by PCR.

### Tetrad dissections

Nup170, Bir1, Htb2 and Mps1 strains resulting from G418 selection after attempted RFP conversion were crossed with strain E223 on YPD. Following selection for diploids and purification, the diploids were sporulated and tetrads dissected on YPD using a MSM dissection microscope (Singer Instruments Ltd). Resulting colonies from 3 days’ growth were replica-plated onto YPD G418 and –HIS media and grown for a further day. Plates were scanned using a desktop flatbed scanner (Epson V750 Pro, Seiko Epson Corporation).

### Marker switching to other fluorophores

Sequences encoding fluorescent proteins azurite, CFP and YFP were obtained as GeneArt Strings DNA fragments (ThermoFisher Scientific) and cloned as derivatives of pRFP-template to form new template plasmids. These plasmids (pAzurite-template, pCFP-template and pYFP-template) in combination with pCas9;GFP1-guide were transformed into the UDS and underwent the same media transfer procedure on agar plates illustrated in Fig. 4a, except with the initial step performed on YPD rather than YP Raffinose. Following this, colonies were transferred to YPD G418 then checked with fluorescence microscopy and PCR. PCR products were then sequenced to check the insertion.

### Dual targeting

Experimental design is illustrated in Fig. 8a. The CAN1-guide sequence from plasmid pCAN1-guide was incorporated into plasmid pCas9;GFP1-guide in order to create a construct containing both guides, on either side of the *NAT* selectable marker.

A new sequence was designed to include ~ 50 bp homology to the start and end of the *CAN1* gene on either side of a *HYG* gene, the whole sequence of which is then flanked by *CAN1* protospacer sequences including the PAM sequences, creating endonuclease targets for the formation of linear constructs upon *CAN1*-targeting endonuclease expression. This sequence was assembled into the existing pCas9-GFP1-guide plasmid, forming a single plasmid containing both template sequences, pRFP-template;CAN1-template.

Plasmid pRFP-template;CAN1-template in combination with either plasmid pCas9;GFP1-guide;CAN1-guide plasmid, pCas9;GFP1-guide or pCas9;CAN1-guide were transferred into the strain encoding Htb2-GFP. Cells were grown overnight in –URA Raffinose NAT, then sequentially transferred to YP GAL NAT for two consecutive days before transfer to SC 5-FOA GAL NAT for 1 day. Wash steps were performed in between every transfer. Cells in each culture were quantified then 1500 cells from each culture were plated on YPD, YPD NAT, YPD HYG and YPD NAT HYG. All cultures were set up in three biological replicates. Colonies were counted after 3 days growth and proportions of template DNA integration were calculated as number of colonies formed on the drug selection plates compared to the YPD plates.

## Supplementary information

**Additional file 1 **: **Supplementary Figure 1.** The GFP1 Cas9 target sequence is shown (blue text) with the adjacent PAM site (red text). Three of the closest matches to this sequence in the yeast genome are shown below in the genes YLL012W, YJR102C and YOR131C. Mismatches to the canonical guide sequence are shown as lowercase black text. CATS was performed on GFP strains and non-GFP strains (BY4741) to determine whether these sequences would suffer mutations. After targeting, PCR was used to amplify the regions (shown as green bars) and these sequenced using Sanger sequencing. No mutations were identified. **Supplementary Table 1.** Plasmids used in this study. Table details the plasmids used in this study. pHT99 is a derivative of pWJ151 2[38] with the *LEU2* marker replaced with NAT resistance gene. n/a indicates ‘not applicable’. **Supplementary Table 2.** Strains used in this study. **Supplementary Table 3.** Summary of tag-switching by plasmid transfer using the UDS. Growth on SC GAL 5-FOA G418 is indicted, which identifies strains that have genomic integration of the *RFP-KAN* template DNA. Cells were then checked with microscopy, N/A indicates that no colonies had formed. **Supplementary Table 4.** Counts of colonies formed from dissected spores on -HIS and G418 media. *NUP170* and *BIR1* tag switched strains fail to segregate the *KAN* (G418) marker correctly, whereas *HTB2* and *MPS1* tag switched strains show correct *KAN* (G418) segregation and linkage between the *KAN* (G418) and *HIS* marker genes. **Supplementary Table 5.** Phenotypes from dual targeting. Six colonies from each of three replicates that grew on YPD G418 HYG plates following targeting with a *HYG* cassette to replace *CAN1* and an *RFP-ADH1p-KAN* cassette to replace *GFP.* Growth was tested on –ARG Canavanine to confirm the *CAN1* gene has been disrupted then cells were checked for RFP signal with microscopy. *HTB2-GFP* (*CAN1*+) and the UDS (*can1–100*) were included on the –ARG Canavanine plates as controls.

## Data Availability

All data generated or analysed during this study are included in this published article [and its supplementary information files].

## Abbreviations

5-FOA: 5-fluoroorotic acid
DSB: Double strand break
HDR: Homology-directed repair
HYG: Hygromycin selectable marker
KAN: G418 selectable marker
NAT: Nourseothricin selectable marker
PAM: Protospacer Adjacent Motif
RFP: Red fluorescent protein
SGA: Synthetic Genetic Array
sgRNA: Single guide RNA
SPA: Selective Ploidy Ablation
UDS: Universal Donor Strain
