# Supplementary material for: CATS: Cas9-assisted tag switching. A high-throughput method for exchanging genomic peptide tags in yeast
Source: BMC Genomics. 2020 Mar 10;21:221. doi: 10.1186/s12864-020-6634-9 (PMC7063721; doi:10.1186/s12864-020-6634-9)
Supplement: Supplementary file 1 — Additional file 1 : Supplementary Figure 1. The GFP1 Cas9 target sequence is shown (blue text) with the adjacent PAM site (red text). Three of the closest matches to this sequence in the yeast genome are shown below in the genes YLL012W, YJR102C and YOR131C. Mismatches to the canonical guide sequence are shown as lowercase black text. CATS was performed on GFP strains and non-GFP strains (BY4741) to determine whether these sequences would suffer mutations. After targeting, PCR was used to amplify the regions (shown as green bars) and these sequenced using Sanger sequencing. No mutations were identified. Supplementary Table 1. Plasmids used in this study. Table details the plasmids used in this study. pHT99 is a derivative of pWJ151 2[38] with the LEU2 marker replaced with NAT resistance gene. n/a indicates ‘not applicable’. Supplementary Table 2. Strains used in this study. Supplementary Table 3. Summary of tag-switching by plasmid transfer using the UDS. Growth on SC GAL 5-FOA G418 is indicted, which identifies strains that have genomic integration of the RFP-KAN template DNA. Cells were then checked with microscopy, N/A indicates that no colonies had formed. Supplementary Table 4. Counts of colonies formed from dissected spores on -HIS and G418 media. NUP170 and BIR1 tag switched strains fail to segregate the KAN (G418) marker correctly, whereas HTB2 and MPS1 tag switched strains show correct KAN (G418) segregation and linkage between the KAN (G418) and HIS marker genes. Supplementary Table 5. Phenotypes from dual targeting. Six colonies from each of three replicates that grew on YPD G418 HYG plates following targeting with a HYG cassette to replace CAN1 and an RFP-ADH1p-KAN cassette to replace GFP. Growth was tested on –ARG Canavanine to confirm the CAN1 gene has been disrupted then cells were checked for RFP signal with microscopy. HTB2-GFP (CAN1+) and the UDS (can1–100) were included on the –ARG Canavanine plates as controls. [file 12864_2020_6634_MOESM1_ESM.pdf]

Supplementary Figure 1

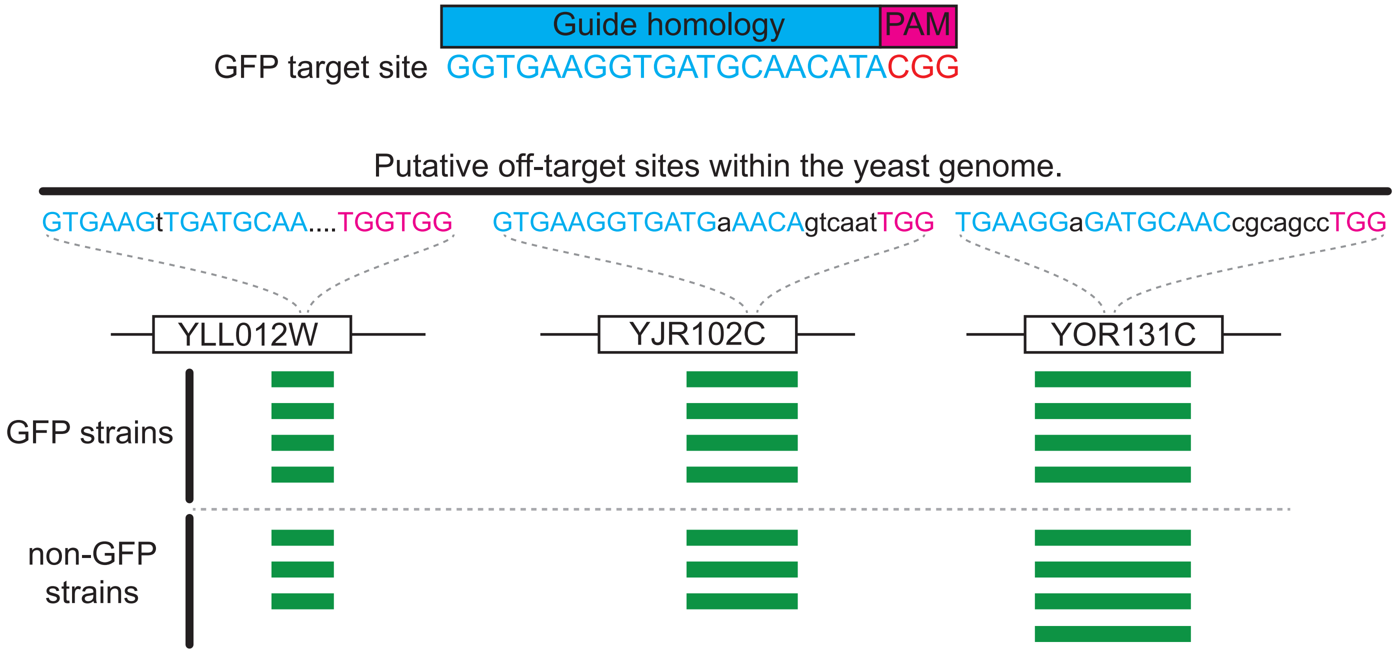

Supplementary Table 1

**Plasmid name**	**Laboratory reference name**	**Genotype**	**Yeast Selection**	**Guide sequence**	**Source**
pCas9;LEU2	p415	p415 *GALLpr-CAS9-CYC1t*	LEU		DiCarlo et al., 2013, Addgene plasmid #43804
pCAN1-guide	p426	*SNR52p-gRNA.CAN1.Y-SUP4t*	URA		DiCarlo et al., 2013, Addgene plasmid #43803
pDead-Cas9	pHT391	*GalLPR-dCAS9-CYC1t*	LEU		This study
pCas9;CAN1-guide	pHT590-2	*GalL-CAS9-ADH1t SNR52p-gRNA.CAN1.Y-SUP4t*	NAT		This study
empty vector	pHT99	pCUP1 (empty)	NAT		Olafsson & Thorpe, 2015
pCas9;GFP1-guide	pHT592-3	GalLp-Cas9-ADH1t SNR52p-gRNA.GFP1.Y-SUP4t	NAT	GGTGAAGGTGATGCAACATA	This study
pCas9;GFP2-guide	pHT594-1	GalL-Cas9-ADH1t SNR52p-gRNA.GFP2.Y-SUP4t	NAT	GTAGTGACAAGTGTTGGCCA	This study
pCas9;GFP3-guide	pHT596-1	GalL-Cas9-ADH1t SNR52p-gRNA.GFP3.Y-SUP4t	NAT	GGTTGTCTGGTAAAGGACA	This study
pUC19-RFP-template	pHT613	SnaBI.GFPstart-RFP-ADH1p-KAN-GFPend.SnaBI	n/a		This study
pRFP-template	pHT642-1	GFPstart-RFP-ADH1p-KAN-GFPend	URA/G418		This study
pCas9	pHT571-7	GalL-Cas9-ADH1t	NAT		This study
pAzurite-template	pHT702-3	GFPstart-Azurite-ADH1p-KAN-GFPend	URA/G418		This study
pCFP-template	pHT703-3	GFPstart-CFP-ADH1p-KAN-GFPend	URA/G418		This study
pYFP-template	pHT704-3	GFPstart-YFP-ADH1p-KAN-GFPend	URA/G418		This study
pCas9;GFP1-guide;CAN1-guide	pHT697-2	*GalL-Cas9-ADH1t SNR52p-gRNA.GFP1.Y-SUP4t SNR52p-gRNA.CAN1.Y-SUP4t*	NAT		This study
pRFP-template;CAN1-template	pHT727-3	*GFPstart-RFP-ADH1pr-KAN-GFPend CANstart-HYG-CAN1end*	URA/G418		This study

Supplementary table 2

Strain	reference name	Genotype	Strain background	Source
PT141	E166	*MAT**a** trp1-1 LYS2 ADE2 leu2-3,112 his3-11,15 ura3-1 CAN1 RAD5*	W303	This study
GFP collection		*MAT**a** his3Δ1 leu2Δ0 met15Δ0 ura3Δ0 X-GFP::HIS6MX*	BY4741	Huh et al., 2003
UDS	W8164-2B	*MATα trp1-1 his3-11,15 leu2-3,112 ura3-1 can1-100 RAD5 MET17 ADE2 LYS2 CEN1-16::Gal-Kl-URA3*	W303	Reid et al., 2011
GFP collection control strain	BY4741	*MAT**a** his3Δ1 leu2Δ0 met15Δ0 ura3Δ0*	BY4741	Brachmann et al., 1998
E223		*MATα ADE2 bar1::LEU2 TRP1 lys2 RAD5*	W303	Reid et al., 2011

Supplementary Table 3

**Strain**	**Plasmids**	**Growth on SC GAL 5'FOA G418**	**Microscopy observation**
HTA2-GFP	pCas9;GFP1-guide and pRFP-template	Yes	RFP
	pCas9 and pRFP-template	No	N/A
RPA49-GFP	pCas9;GFP1-guide and pRFP-template	Yes	RFP
	pCas9 and pRFP-template	No	N/A

Supplementary Table 4

			No. growing on:		
Strain	No. spores dissected	No. viable spores	G418	HIS-	Both
NUP170	40	35	2	16	1
BIR1	40	39	0	20	0
HTB2	20	20	10	10	10
MPS1	16	16	8	8	8

Supplementary Table 5

Strain	Plasmids	Colony	Growth on -ARG Canavanine	Microscopy observation
HTB2-GFP	pCas9;GFP1-guide;CAN1-guide and pRFP-template;CAN1-template	1	Yes	RFP
HTB2-GFP	pCas9;GFP1-guide;CAN1-guide and pRFP-template;CAN1-template	2	Yes	RFP
HTB2-GFP	pCas9;GFP1-guide;CAN1-guide and pRFP-template;CAN1-template	3	Yes	RFP
HTB2-GFP	pCas9;GFP1-guide;CAN1-guide and pRFP-template;CAN1-template	4	Yes	RFP
HTB2-GFP	pCas9;GFP1-guide;CAN1-guide and pRFP-template;CAN1-template	5	Yes	RFP
HTB2-GFP	pCas9;GFP1-guide;CAN1-guide and pRFP-template;CAN1-template	6	Yes	RFP
HTB2-GFP	pCas9;GFP1-guide;CAN1-guide and pRFP-template;CAN1-template	7	Yes	RFP
HTB2-GFP	pCas9;GFP1-guide;CAN1-guide and pRFP-template;CAN1-template	8	Yes	RFP
HTB2-GFP	pCas9;GFP1-guide;CAN1-guide and pRFP-template;CAN1-template	9	Yes	RFP
HTB2-GFP	pCas9;GFP1-guide;CAN1-guide and pRFP-template;CAN1-template	10	Yes	RFP
HTB2-GFP	pCas9;GFP1-guide;CAN1-guide and pRFP-template;CAN1-template	11	Yes	RFP
HTB2-GFP	pCas9;GFP1-guide;CAN1-guide and pRFP-template;CAN1-template	12	Yes	RFP
HTB2-GFP	pCas9;GFP1-guide;CAN1-guide and pRFP-template;CAN1-template	13	Yes	RFP
HTB2-GFP	pCas9;GFP1-guide;CAN1-guide and pRFP-template;CAN1-template	14	Yes	RFP
HTB2-GFP	pCas9;GFP1-guide;CAN1-guide and pRFP-template;CAN1-template	15	Yes	RFP
HTB2-GFP	pCas9;GFP1-guide;CAN1-guide and pRFP-template;CAN1-template	16	Yes	RFP
HTB2-GFP	pCas9;GFP1-guide;CAN1-guide and pRFP-template;CAN1-template	17	Yes	RFP
HTB2-GFP	pCas9;GFP1-guide;CAN1-guide and pRFP-template;CAN1-template	18	Yes	RFP
HTB2-GFP	None		No	
UDS	None		Yes	
